# Laryngitis—Tracheotomy

**Published:** 1831

**Authors:** 


VIII.

LARYNGITIS—TRACHEOTOMY.

DR. ADAM HUNTER, Physician to the Leeds General Infirmary, has published an interesting case of this kind in a late Number of the North of England Journal, which we shall here abbreviate.

*Case.* Betty Fieldhouse, aged 30, was admitted into the Leeds Infirmary, 14th May, 1830. She was emaciated, and the mother of five children. Sixteen months previously, (during a confinement) she was attacked with sore throat, which, after some time, increased in severity, and was attended with difficulty of breathing and swallowing. When received, her eyes were hollow—respiration wheezing—dysphagia—tumour or thickening behind the epiglottis—weak voice—frequent cough—mucous expectoration. Leeches, blisters, iodine, and sarsaparilla were prescribed. Afterwards a grain of calomel was ordered every night. No benefit appears to have accrued from the remedies, and on the 26th of May, we find the patient threatened with suffocation. On the 27th, in a consultation, tracheotomy was determined on.

*Operation, by Dr. Smith.* "The patient sat in a chair, the head being inclined backwards and held by an assistant; a perpendicular incision, an inch and a half in length, was made, by means of a fine small scalpel, upon the triangular space between the thyroid and cricoid cartilages, which was punctured with a bistoury. She took a deep inspiration at this moment, when a few drops of blood were drawn into the trachea, which produced such violent spasm as to prevent the progress of the operation for three or four minutes. Upon its subsidence, one or two veins were secured to prevent confusion; and her head placed a little forward, as she breathed easiest in that posture. The bistoury was again introduced, making a crucial incision, and cutting away the flaps. The opening not being sufficiently large, a small portion of the upper part of the cricoid cartilage was scooped out. Her countenance brightened with an expression indicative of great relief. She swallowed two or three tea-spoonfuls of tea immediately afterwards, the first food of thirty-six hours; and by signs, expressed herself much relieved. An assistant watched her for three hours, during two of which she slept calmly, being the first sleep she had enjoyed for forty-eight hours.

6, p.m.—The orifice becoming choked, a coagulum was removed, and the sterno-thyroid muscle being drawn aside she breathed with ease.—To keep the orifice open, a probe was bent in the form of a speculum oculi, and retracting the muscle it was bound there by straps of Emp. Adhesiv. This succeeded only for a short time. Tea taken in two or three spoonfuls, passed entirely into the trachea; which produced much coughing. Milk thickened with flour to be administered per rectum every two hours.

10, p.m.—A short canula was introduced with the inferior part cut away, and held in situ by Emp. Adhesiv. Rests comfortably from its use. Before the operation, the pulse was quick and frequent, it afterwards improved and remained at 100.

28th.—2, a.m.—She was asleep: at 3 she awoke—the canula becoming choked, it was removed and cleaned, and the upper part cut away—she slept until 6½, a.m. when she was tolerably easy—got a few drops of tea down the œsophagus. The enemata continued.

11, a.m.—Internal orifice becoming contracted, a canula of larger caliber, cut similar to the former one, was introduced, the points being first made conical, and expanded afterwards further than the caliber of its central portion by means of a pair of dressing forceps, thus retaining itself in its situation, as the dressings being kept constantly loose by the discharge, would not adhere. Breathes easier, continues to slumber.—Expectoration has been free since the operation, although, upon a forcible expiration, the mucus is partially expelled, when the following inspiration retracts it.

4, p.m.—Much exhausted—pulse feeble at 130, skin cool—tongue moist. Passing the finger down the anterior part of the throat, the epiglottis felt thickened and elevated, the rima glottidis thickened, and posteriorly an elevation turning back over the œsophagus. 7, p.m.—Has swallowed nothing during the last 24 hours."

We need not pursue the narrative through the minute details. The patient lived till the 10th of June, when she sunk exhausted, and unable to expel the mucus from the trachea.

### Post-mortem Examination.

"The larynx completely closed and impervious, apparently the consequence of chronic inflammation, so that respiration must have been carried on entirely by the artificial opening during the last ten days. The œsophagus, about two inches downwards, is adherent to the back part of the trachea; the anterior and posterior parts of the œsophagus also adhere to each other so closely, that a probe cannot be passed upwards or downwards. The œsophagus below the stricture was rather diminished in caliber, although perfectly healthy. Upon slitting it open behind, the fore-part of the Pharynx was found to be ulcerated in several places, with many deep fissures on its anterior part filled with mucus. One in particular, situated close to where the stricture was greatest, appeared to communicate with the Trachea; but upon examination was found to be a cul-de-sac. When the Epiglottis is depressed upon the Larynx, an open triangular space is left to the back part, the portions upon which it rests being thickened and elevated by inflammation.* Upon refer-

* "This, along with the stricture of the Œsophagus, will account for the ready passage the food found into the Trachea, and out at the artificial opening between the Thyroid and cricoid Cartilages."

ring to the accompanying plate, it will be found that, just below the orifice marked as a cul-de-sac, the diameter of the œsophagus was not sufficient to admit even a probe."

